# Palladium (0) nanoparticles distributed on lanthanum (III) oxide as an effective catalyst for the methanolysis of hydrazine-borane to produce hydrogen

**DOI:** 10.55730/1300-0527.3646

**Published:** 2024-01-03

**Authors:** Adem RÜZGAR, Lokman ŞENER, Yaşar KARATAŞ, Mehmet GÜLCAN

**Affiliations:** Department of Chemistry, Faculty of Science, Van Yüzüncü Yil University, Van, Turkiye

**Keywords:** Hydrazine-borane, hydrogen, methanolysis, nanoparticles, palladium

## Abstract

Pd (0) nanoparticles (NPs) distributed on lanthanum (III) oxide were ex situ generated from the reduction of Pd^2+^ ions using NaBH_4_ as reducing agent. The Pd/La_2_O_3_ displayed good catalytic activity in H_2_(g) releasing from the hydrazine-borane (HB) methanolysis reaction and it was identified by advanced techniques. Pd/La_2_O_3_ was found to be an active catalyst procuring three equiv. H_2_(g) per mole of HB. The results from TEM images represent the formation of Pd (0) NPs with an average particle size of 1.94 ± 0.1 nm on the surface of La_2_O_3_. Moreover, Pd/La_2_O_3_ with various Pd loadings were prepared and tested as catalyst in the methanolysis reaction to find the optimum metal loading on La_2_O_3_ support. The highest H_2_ formation rate was achieved with 3.0 wt% Pd. Pd/La_2_O_3_ catalyst exhibited a turnover frequency (TOF) value of 24.4 mol H_2_ mol Pd^−1^ min^−1^ in the reaction conditions. Additionally, the effect of different catalyst concentrations and temperatures on the reaction kinetics for the methanolysis of HB catalyzed by Pd/La_2_O_3_.

## 1. Introduction

Today, one of the primary objectives for all developed countries is to achieve sustainable economic growth and development. This shared goal has led to an exponential increase in the world’s energy needs day by day. This rising demand for energy has unpredictably driven up the reliance on fossil fuels over the past few centuries. The use of fossil fuels not only results in water, soil, and air pollution but also poses a significant threat to human health. Moreover, it contributes to multifaceted global issues such as global warming, impacting the entire planet. Apart from these detrimental aspects, the production, transportation, and storage of fossil fuels are both expensive and hazardous. In response to these challenges associated with fossil fuels, numerous research and practical studies are underway, focusing on “alternative energy sources” [1–3]. Among these, hydrogen energy, often referred to as the energy source of the new century, stands out as a leading subject in these investigations [4–12].

Hydrogen, the lightest element, is a colorless, odorless, tasteless, flammable diatomic gas under standard conditions. One of the most important features of hydrogen gas is that it can be obtained from both inexhaustible (renewable) and fossil (nonrenewable) fuels. In addition, during the production and consumption of hydrogen; harmful wastes and chemical transformations encountered in production/consumption processes of fossil fuels are not observed. Therefore, hydrogen emerges as an energy source offering several sought-after features, including environmental friendliness, health benefits, economic viability, and ease of production [13–16]. Despite these advantages, when the quantitative distribution of energy sources that are widely used today is examined, it becomes apparent that hydrogen has not yet achieved widespread adoption as an energy source. When analyzing the obstacles hindering the widespread use of hydrogen as a substitute for conventional energy sources, it becomes evident that the most significant challenges revolve around creating efficient, safe, and cost-effective storage and transportation capabilities [17–18]. Additionally, with growing interest in reducing greenhouse gas emissions, renewable energy sources are gaining momentum as clean options for producing hydrogen as an energy carrier, devoid of carbon emissions. Renewable hydrogen serves as a bridge between renewable energy sources and the modernization of energy supply, transportation, industry, and renewable energy exports. Given hydrogen’s versatility in direct use as fuel (pure H_2_ or fuel mixtures) and conversion into other liquid or gaseous fuels, it can be argued that a hydrogen-based energy system offers greater durability compared to traditional fossil fuel-based systems [19–21].

Hydrogen can be stored in various forms, including high-pressure gaseous, liquid, metal, or chemical hydrides. Storing hydrogen as a gas in high-pressure vessels or as a liquid at cryogenic temperatures poses serious environmental, safety, and cost issues. These challenges have spurred increased research and applications related to materials capable of storing hydrogen in the solid phase. Materials such as carbon nanotubes [22–24], metal hydrides and amine boranes [25–26], nanomaterials [27], and organometallic structures [28] are among those explored. Among these materials, amine-boranes and their derivatives have come to the forefront due to their stable structures, high hydrogen content per unit volume, being economical and environmentally friendly and have been the subject of many studies [29–35]. Within these derivative compounds, hydrazine-borane (N_2_H_4_BH_3_, HB) has captured researchers’ attention as a promising boron-based compound, boasting high hydrogen content (15.4 wt%) with four protic (N-H) hydrogens compared to three hydrogen (B-H) hydrides [36–44]. Research on HB has revealed its advantages, such as being solid at room temperature, water solubility, environmental nontoxicity, and stability against spontaneous hydrolysis, similar to other amine-boranes. Additionally, it has been specifically identified to possess a high gravimetric hydrogen density. Hydrogen stored in HB can be released through pyrolysis [45], hydrolysis [46] (1), and methanolysis [47] (2) reactions. However, the most promising approach for hydrogen production from HB involves complete dehydrogenation, wherein the BH_3_ group undergoes hydrolysis, selectively separating N_2_H_4_ into N_2_ and H_2_ (3). Nevertheless, to maximize the efficiency of HB as a hydrogen storage material, undesired decomposition of N_2_H_4_ into NH_3_ and N_2_ (4) should be avoided. This undesired catalytic decomposition not only reduces the produced amount of H_2_ but also generates NH_3_, which can have a toxic effect on fuel cells. Studies have shown that these reaction pathways largely depend on catalyst design and reaction conditions. In this context, using a basic catalyst support and operating in a highly alkaline solution environment selectively advances the catalytic reaction in the desired direction [48–49].


(1) $${\text{N}}_{2}{\text{H}}_{4}{\text{BH}}_{3}\;(\text{s})+3{\text{H}}_{2}\text{O }(\text{l})\to {\text{N}}_{2}{\text{H}}_{4}\;(\text{aq})+{\text{H}}_{3}{\text{BO}}_{3}\;(\text{l})+3{\text{H}}_{2}\;(\text{g})$$

(2) $${\text{N}}_{2}{\text{H}}_{4}{\text{BH}}_{3}\;(\text{s})+4{\text{CH}}_{3}\text{OH }(\text{l})\to {\text{N}}_{2}{\text{H}}_{5}\text{B }{\left({\text{OCH}}_{3}\right)}_{4}\;(\text{aq})+3{\text{H}}_{2}\;(\text{g})$$

(3) $${\text{N}}_{2}{\text{H}}_{4}\;(\text{aq})\to {\text{N}}_{2}\;(\text{g})+2{\text{H}}_{2}\;(\text{g})$$

(4) $$3{\text{N}}_{2}{\text{H}}_{4}\;(\text{aq})\to 4{\text{NH}}_{3}\;(\text{g})+{\text{N}}_{2}\;(\text{g})$$

Two main problems encountered during the hydrolysis of amine-borane (AB) are that the hydrogen stored in the NH_3_ group within the structure cannot be completely liberated under hydrolysis conditions, and NH_3_ can have a poisoning effect on the catalyst at high substrate concentrations. On the other hand, these problems encountered during the hydrolysis of AB are not experienced in the hydrogen production from HB. In addition to considering the mechanistic properties of the catalytic reaction, it is possible to find out the hydrogens in the N_2_H_4_ group in the structure of HB in the specifically conducted hydrogen generation processes. In these selectively carried out reactions, dehydrogenation of 1 mole of N_2_H_4_BH_3_ yields H_2_ and N_2_ in a mole ratio of 5:1. Due to the aforementioned advantages, studies aiming to produce hydrogen from HB through both hydrolysis and methanolysis have gained attention. The hydrolysis reaction can be conducted under relatively milder conditions, yielding satisfactory hydrogen evolution efficiency. However, there is an issue related to the inherent instability of HB against self-hydrolysis. In the alcoholysis reaction of HB, it has been observed that HB is more stable in methanol and does not undergo self-methanolysis. This suggests that effective hydrogen evolution from HB can be achieved in the presence of a suitable catalyst [50–52].

Research aimed at developing hydrogen production processes from HB via methanolysis has highlighted the indispensable need for suitable nanocatalysts to achieve fast, efficient, and controllable reaction environments. Nanocatalysts possess distinct physical and chemical properties compared to metal ingots, making them of great interest in recent years [53–60]. Metal nanocatalysts can show high efficiency and selectivity in catalytic reactions where metal ingots are not active. The main reason for the high catalytic efficiency they show in the reactions is the increase in the number of catalytically effective atoms on their surfaces despite the reduction in particle sizes [61–64].

Despite the positive properties mentioned above, metal nanoparticles (NPs) are kinetically unstable against the formation of metal nuggets and aggregation due to their high surface energies. Such agglomerations that will occur in the catalyst system can shorten the catalyst’s lifespan and reduce reaction efficiency. To prevent this undesirable situation, in other words, to keep the nanocatalysts colloidally stable in the solution environment, suitable stabilizing groups have been used. Carbon-based materials, metal oxides, metal-organic lattice structures, polymers, and zeolites are among these groups [65–68].

The key parameters in HB dehydrogenation include the presence of a selective and active catalyst capable of decomposing both the BH_3_ and N_2_H_4_ groups within the HB structure, as well as the development of support systems to prevent catalyst aggregation. The objective is to create efficient catalyst systems by combining rhodium, ruthenium, iridium, nickel, platinum, palladium with metals such as cobalt and copper, along with distinctive support supplies [69–79].

Rare earth elements (REE) are widely recognized for their unique chemical structures and exceptional catalytic, magnetic, and electronic properties, making them valuable in various industries and biotechnological applications. Lanthanum oxide (La_2_O_3_), among REE oxides, finds applications in sensors, electronic and luminescent devices, fuel cells, magnetic data storage, antimicrobial agents, catalysis, automobiles, water treatment, phosphate removal, and biomedical research [80–83]. With a bandgap ranging from 4.3 eV to 5.8 eV, La_2_O_3_ is a semiconductor material with the largest bandgap among REE oxides. It crystallizes in a hexagonal system structure with the *P3m1* space group and has a low photon energy of about 400 cm^−1^, making it an excellent host matrix when doped with metals or other metal oxides [84–86].

In our study, we prepared La_2_O_3_-supported palladium nanoparticles (Pd NPs)—Pd/La_2_O_3_—for the methanolysis-based dehydrogenation of HB, and assessed the efficiency of the catalyst under mild reaction conditions. Characterization using powder X-ray diffraction (P-XRD), X-ray photoelectron spectroscopy (XPS), scanning electron microscopy (SEM), SEM-elemental mapping, transmission electron microscopy (TEM), high-resolution TEM (HRTEM), and inductively coupled plasma optical emission spectroscopy (ICP-OES) confirmed that Pd (0) NPs were well spread on the surface of La_2_O_3_. Pd/La_2_O_3_ exhibited good catalytic activity in the methanolysis of HB, with an initial turnover frequency (TOF) of 24.4 mol H_2_ mol Pd^−1^ min^−1^ at 25 ± 0.1 °C, marking it as the second heterogeneous catalytic system ever reported for HB methanolysis. Furthermore, stability and durability experiments revealed that the Pd/La_2_O_3_ catalyst maintained its catalytic efficiency and activity to a great extent even after the 5th cycle, highlighting its high reusability and stability as a heterogeneous catalyst for HB methanolysis.

## 2. Experimental

### 2.1. Synthesis of La_2_O_3_ supported Pd (0) NPs

To synthesize the Pd/La_2_O_3_ catalyst, we first prepared a 5-mL solution containing 3% by weight Pd metal (11.22 mg, 42.1 μmol) using Pd(NO_3_)_2_·2H_2_O salt. To this solution prepared using deionized water, 150 mg of La_2_O_3_ was added and the mixture was stirred at 750 rpm for 3 h. After stirring, an excess amount of NaBH_4_ was added dropwise to reduce Pd(II) to Pd(0), (with an estimated metal/NaBH_4_ molar ratio of 15). Once the bubbling completely ceased, indicating complete reduction of Pd(II) to Pd(0), the resulting product was filtered and washed with deionized water (3 × 20 mL) for purification. Finally, the Pd/La_2_O_3_ catalyst obtained from these processes was dried under appropriate conditions (Scheme).

### 2.2. Catalytic activity in the HB methanolysis

The main method used to assess the catalytic efficiency of Pd/La_2_O_3_ in the methanolysis of HB is to measure the gas formation rate. For this purpose, it was attempted to determine the catalytic efficiency by volumetrically measuring gas formation rate in different parameters. A graduated glass cylinder, resembling a gas burette, was used to determine the volume of H_2_ gas produced during HB methanolysis. This involved periodically monitoring the displacement of water in the graduated cylinder due to the released H_2_ gas [87]. Temperature regulation was achieved by circulating water in the system, where a 50 mL single-necked reaction balloon was positioned on a magnetic stirrer. Gas volume released was determined by measuring the volumetric displacement of water in the graduated cylinder, placed on a jacketed Schlenk, designed for temperature regulation. The necessary graphs were generated accordingly. Following the experimental setup, 100 mg (25.8 μmol, 4.69 mM) of Pd/La_2_O_3_ catalyst was placed on the Schlenk apparatus set at 298 K, and then 4 mL of dried methanol was added. The mouth of the jacketed Schlenk, whose temperature was controlled by means of a circulator, was sealed to prevent gas escape, and the system was stirred for about 15 min until it reached equilibrium. Subsequently, 46 mg of HB dissolved in 1 mL of dried methanol was injected into the experimental setup, maintaining a fixed stirring speed of 750 rpm. Finally, the volume (mL) of gas released due to HB methanolysis was recorded against time (min), and the corresponding graphs were plotted.

The methanol used in the experimental studies was dried through the following procedure: Methanol was dried by heating on iodine-activated magnesium with a magnesium loading of 0.5–5.0 g/L [88]. Small pieces of magnesium metal were introduced into methanol and refluxed for 2–3 h under a nitrogen atmosphere. The dry methanol was then cooled to room temperature and stored in an inert environment.

### 2.3. Determination of most effective Pd loading for Pd/La_2_O_3_

Various Pd-loaded samples (1.0%–4.0% by weight) were prepared to determine the most effective Pd loading for Pd/La_2_O_3_. These samples, each weighing 100 mg, were tested for H_2_ gas release from the methanolysis of HB (46 mg, 200 mM HB) at 298 K. The highest H_2_ formation rate was achieved for the catalyst sample containing 3.0 wt% Pd. Subsequently, the catalyst sample with 3.0 wt% Pd loading was used for further catalytic reactions.

### 2.4. Durability experiments

To assess the catalytic reusability and durability of the Pd/La_2_O_3_ catalyst in the methanolysis of HB at room temperature, a specific amount of the catalyst was used to prepare a 5-mL solution (100 mg, 25.8 μmol, 4.69 mM). This solution, along with 200 mM HB, was employed in a series of experiments. After each experiment, once the targeted reaction was complete, an equal amount of HB was reintroduced to the reaction medium. The durability data for the Pd/La_2_O_3_ catalyst in the methanolysis of HB was recorded as a percentage.

## 3. Results and discussion

P-XRD, XPS, TEM, HRTEM, SEM, and ICP-OES analyses were conducted on the Pd/La_2_O_3_ catalyst to assess its structural and morphological properties. Initially, ICP-OES analysis was utilized to determine the distribution of Pd metal content on La_2_O_3_, revealing a Pd content of 2.7 ± 0.1 wt%. It is important to note that the theoretical metal content of the Pd/La_2_O_3_ catalyst was designed to contain 3.0 wt% Pd.

**Figure 1:**
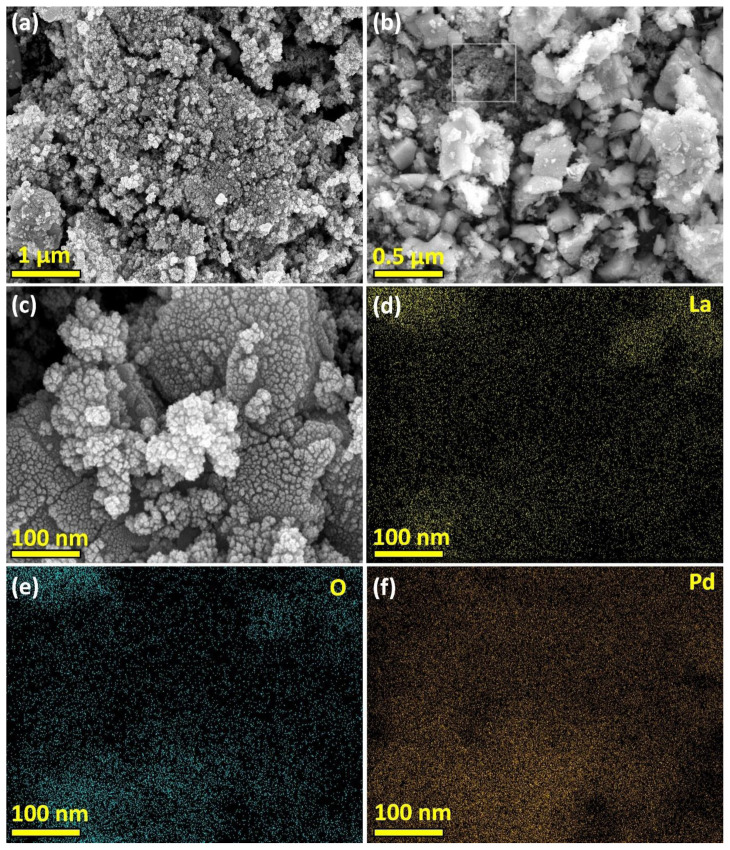
SEM image at different scales (a–c), along with corresponding elemental mapping La (d), O (e), and Pd (f) on the surface of the Pd/La_2_O_3_ catalyst.

SEM analysis was performed to examine the distribution of Pd NPs on the La_2_O_3_ surface, which served as a support, and to characterize the surface morphology (Figures 1a–1c). To achieve the desired image quality, the sample of the Pd/La_2_O_3_ catalyst was coated with gold for approximately 100 s. SEM images and corresponding elemental mapping of Pd/La_2_O_3_ confirmed the presence of La, O, and Pd elements and the uniform dispersion of Pd NPs within the Pd/La_2_O_3_ catalyst. On the other hand, Pd exhibited a homogeneous distribution similar to other elements but with a lower density. This is also associated with the contribution of Pd (0) NPs at lower concentrations compared to other elements. As confirmed by the SEM elemental mapping in the obtained SEM images, it is noticed that Pd NPs were extremely uniformly distributed on La_2_O_3_ surface (Figures 1d–1f).

**Figure 2:**
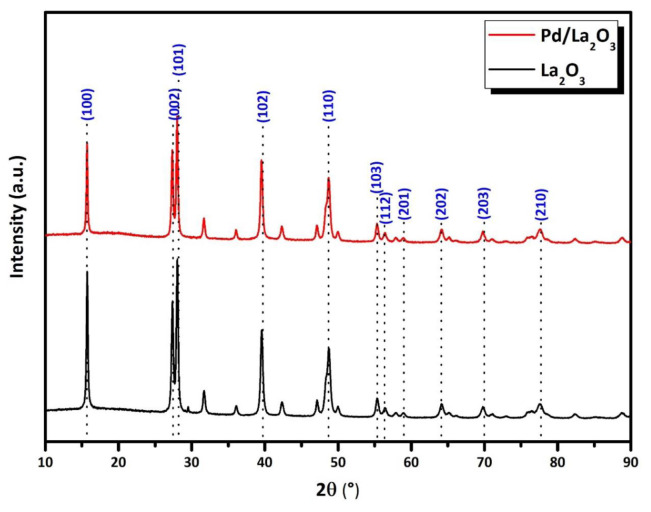
P-XRD patterns of the Pd/La_2_O_3_ catalyst and La_2_O_3_ solid support at 2θ = 10°–90°.

The preservation of an intact La_2_O_3_ structure after the deposition of Pd NPs was confirmed by the Bragg peaks of the La_2_O_3_ support shown in the P-XRD patterns of the La_2_O_3_ structure and the Pd/La2O3 catalyst (Figure 2). The major XRD diffraction peaks of La_2_O_3_ corresponded well with the hexagonal La_2_O_3_ phase (JCPDS: 05–0602). The Bragg diffraction peaks at 2θ values of 15.7°, 27.2°, 28.2°, 39.5°, 48.7°, 55.4°, 56.5°, 59.1°, 64.1°, 69.7°, and 77.7° are respectively indexed to the crystal planes (100), (002), (101), (102), (110), (103), (112), (201), (202), (203), and (210) of La_2_O_3_ [89–90]. The absence of Bragg peaks attributable to Pd NPs indicated a low (<5% by weight) loading of Pd NPs on the host La_2_O_3_ surface. Additionally, some changes in charge distribution and electrostatic fields were observed due to the interaction of Pd NPs and electrophilic structures on the surface with framework atoms. These changes were largely attributed to the decrease in the intensity of Bragg peaks observed in the Pd/La_2_O_3_ catalyst [64].

**Figure 3:**
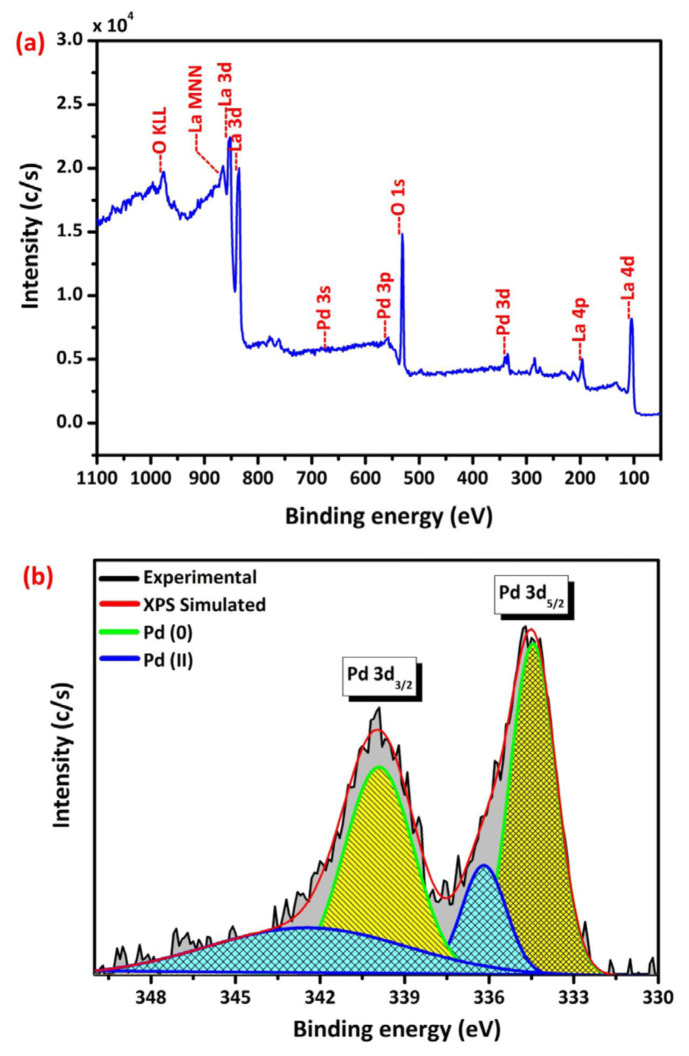
Survey scan (a) and Pd 3d core level XPS spectra of Pd/La_2_O_3_ catalyst.

XPS analysis was employed to identify the elements present in the Pd/La_2_O_3_ catalyst and elucidate its surface characteristics. The survey analysis of the Pd/La_2_O_3_ catalyst in Figure 3a revealed the presence of the Pd element along with the framework elements of La_2_O_3_. High-resolution spectrum of Pd 3d bands displayed two prominent peaks at 334.4 and 339.9 eV corresponding to metallic Pd 3d_5/2_ and Pd 3d_3/2_, respectively [32,64,91]. This indicates reduction of Pd^2+^ ions used as a precursor and the existence of metallic Pd in the Pd (0) form within the catalyst sample. Additionally, small peaks observed at 342.4 eV and 336.2 eV were associated with Pd–O 3d_3/2_ and Pd–O 3d_5/2_ bonds, respectively [92].

**Figure 4:**
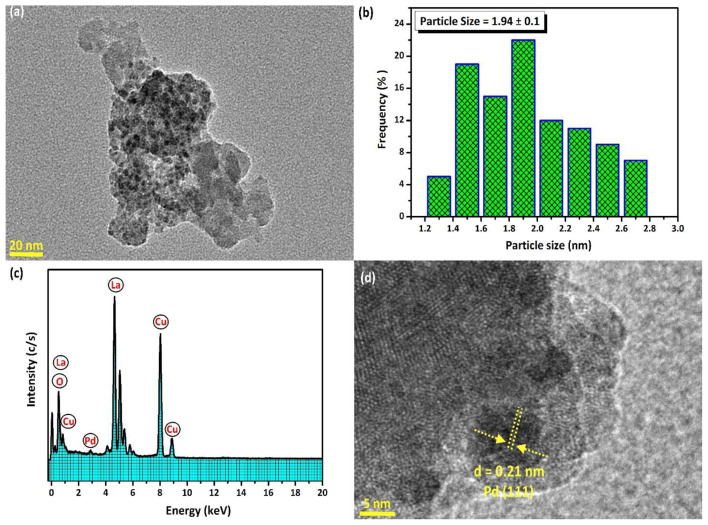
(a) TEM image of Pd/La_2_O_3_ (2.7 ± 0.1 wt % Pd) catalyst), (b) size distribution histogram for Pd (0) NPs, (c) TEM–EDX spectrum, and (d) HRTEM image of the Pd/La_2_O_3_ catalyst.

**Figure 5:**
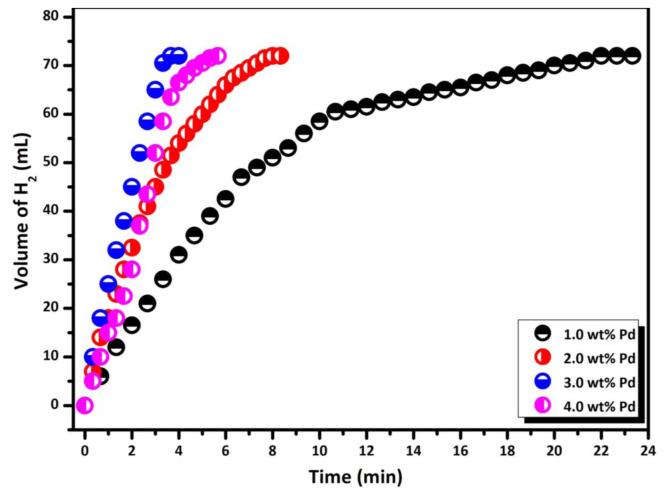
Graphs of released volume of gas vs. time for 200 mM HB methanolysis in different Pd loadings (1.0, 2.0, 3.0, 4.0 wt% Pd) at 298 K.

TEM images of the Pd/La_2_O_3_ catalyst with 3.0 wt% Pd loading are presented in Figure 4. These images reveal that Pd (0) NPs are uniformly distributed on the surface of La_2_O_3_ at various magnifications. A particle size histogram was generated by counting more than 100 nontouching particles, determining the mean diameter of Pd (0) NPs as 1.94 ± 0.1 nm. The TEM–EDX spectrum of the Pd/La_2_O_3_ catalyst (2.7 ± 0.1 wt% Pd) in Figure 5c confirms the presence of La, O, and Pd as the only elements, consistent with XPS analysis in Figure 3a. Additionally, the HRTEM image of the Pd/La_2_O_3_ catalyst indicates a d-spacing of 0.21 nm corresponding to the Pd (111) lattice plane (Figure 4d) [32,64,91].

Before conducting catalytic activity tests and detailed kinetic studies, we conducted two significant preliminary investigations. Firstly, the catalytic nature of La_2_O_3_ and Pd/La_2_O_3_ materials in the target catalytic reaction was compared under the same conditions to determine the net reactivity of Pd (0) nanoparticles in the methanolysis of HB. At the end of the experiment, it was determined that the metal-free La_2_O_3_ was not catalytically active in this catalytic conversion (Figure S1). Secondly, Pd-loaded La_2_O_3_ samples containing varying percentages of Pd were prepared, and their catalytic activities were tested in the methanolysis of HB. Figure 5 illustrates the H_2_ evolution graph from HB methanolysis catalyzed by Pd/La_2_O_3_ with different Pd loading ranging from 1.0% to 4.0 wt% Pd. Upon addition of the HB substrate, H_2_ production commenced without an observed induction time and continued until the gas equivalent reached 3.0 in the catalytic methanolysis reaction. The sample with 3.0 wt% Pd loading was established as the most active catalyst for H_2_ generation from HB methanolysis. The H_2_ generation rate (TOF) of the catalyst was calculated as 24.4 mol H_2_ mol Pd^−1^ min^−1^ for Pd/La_2_O_3_ with 3.0 wt% Pd. Higher palladium loadings resulted in a decrease in the surface area of the supporting material and failure to reach active sites of the catalyst. Therefore, Pd/La_2_O_3_ samples with a 3.0 wt% Pd ratio were selected as the optimum ratio for all other experiments.

**Figure 6:**
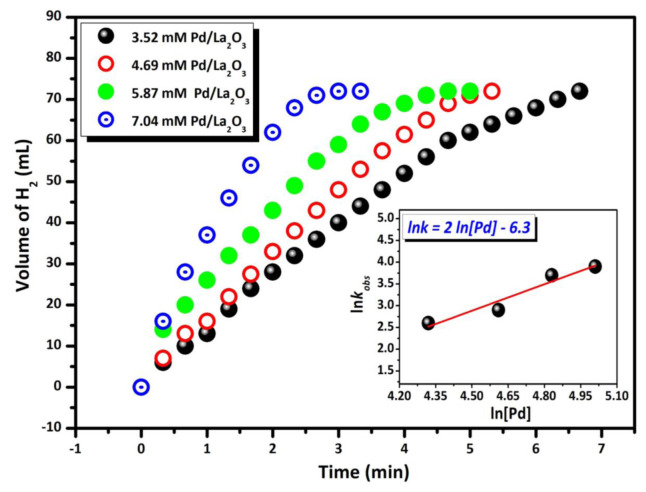
Graphs of the released gas volume vs. time for 200 mM HB methanolysis in different concentrations of Pd/La_2_O_3_ catalyst ([Pd] = 3.52, 4.69, 5.87 and 7.04 mM) at 298 K. Inset: Plot of rate of the hydrogen generation vs. palladium concentration (in logaritmic scale).

Figure 6 illustrates the graphs of H_2_ gas volume (mL) versus H_2_ gas time (min) released when Pd/La_2_O_3_ is utilized as a catalyst in the methanolysis of 200 mM HB at 298 K. Based on the reaction data employing catalysts at varying concentrations, the Pd/La_2_O_3_ catalyst demonstrated outstanding catalytic activity across all reactions. Upon examining the graphs, it is evident that the hydrogen formation processes start without any induction period at all concentrations and continue until all HB in the solution is converted into products, indicating an almost linear methanolysis process of HB. Drawing a graph of Pd concentration versus the rate of hydrogen formation on a logarithmic scale reveals a correlation (see inset of Figure 6), showing that the HB methanolysis process using the Pd/La_2_O_3_ catalyst yields a straight line with a slope of approximately 2.0 concerning the Pd/La_2_O_3_ catalyst concentration.

A significant outcome of the study is the achievement of complete conversion of HB methanolysis within 5 min at 298 K using 2.7 ± 0.1 wt% Pd. The TOF (turnover frequency) value for the Pd/La_2_O_3_catalyst in HB methanolysis was calculated as 24.4 mol H_2_ mol Pd^−1^ min^−1^ at 298 K, representing an important result among catalytic systems employed in HB methanolysis, as summarized in Table. Additionally, the Pd/La_2_O_3_ catalyst represents the second heterogeneous catalytic system used in HB methanolysis.

**Figure 7:**
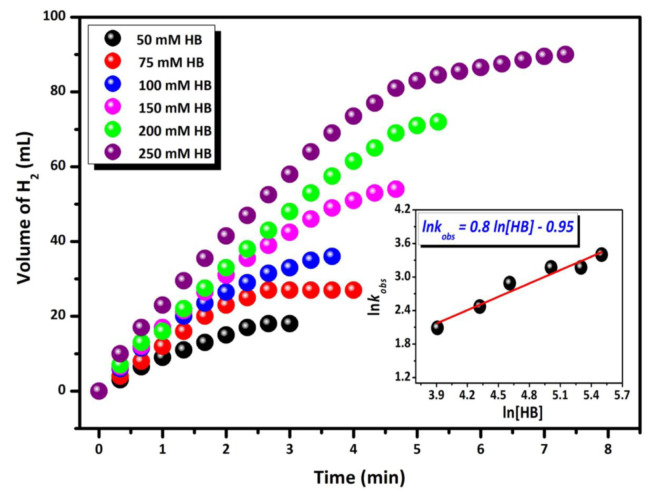
Graphs of released gas volume vs. time for HB methanolysis in different concentrations of HB ([HB] = 100, 150, 200, and 250 mM) at 298 K. Inset: Plot of rate of hydrogen generation vs. concentration of HB (in logaritmic scale).

To assess the effect of HB concentration on the formation rate of H_2_ gas released during catalytic methanolysis, catalytic studies were conducted using various HB concentrations at a constant catalyst concentration ([Pd] = 4.69 mM) and temperature (298 K). The volume of H_2_ gas released in the methanolysis reactions initiated with different HB concentrations was measured at regular intervals, and a volume (mL)/time (min) graph was generated, as shown in Figure 7. Upon examination of the graph, the effect of HB concentration on the reaction’s initial rate appears to be rather limited. The graph of the initial concentration of HB versus the hydrogen production rate yields a line with a slope of ≈0.8 (inset of Figure 7), indicating that HB methanolysis catalyzed by Pd/La_2_O_3_ proceeds kinetically by half-order with HB concentration.

**Figure 8:**
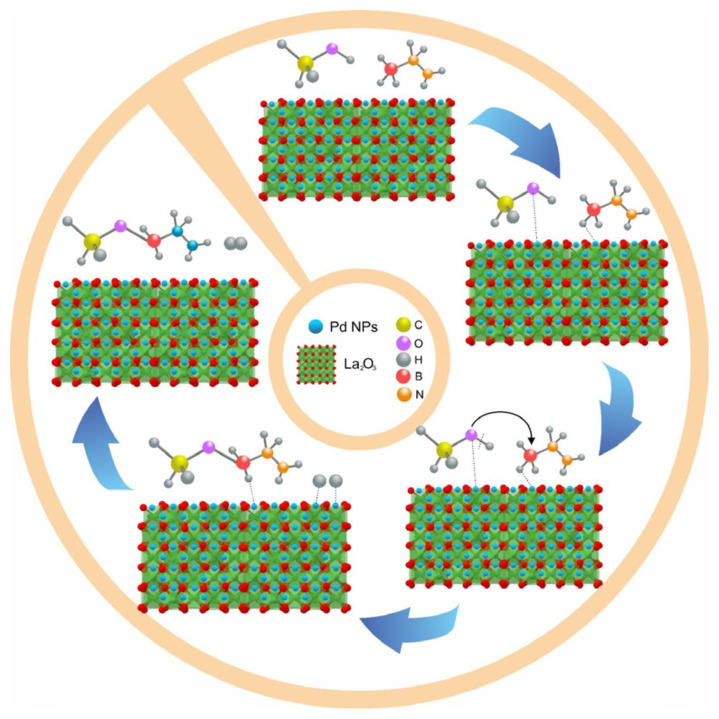
Plausible mechanism of HB methanolysis catalyzed with Pd/La_2_O_3_.

So far, the reaction mechanism of HB methanolysis has not been studied extensively, similar to the AB methanolysis reaction, which has been rarely explored in the literature [95–97]. In this context, it is estimated that the reaction mechanism of HB methanolysis in the presence of the Pd/La_2_O_3_ catalyst can be elucidated as follows, drawing parallels with AB: (i) adsorption and activation of methanol on Pd NPs on La_2_O_3,_ facilitating the absorption of HB molecules; (ii) scission of O–H bond in CH_3_OH to produce H* and *OCH_3_; (iii) attack of OCH_3_ on the B atom in HB, leading to the formation of N_2_H_4_BH_2_–OCH_3_ and another H; (iv) combination of two H to form one H_2_ molecule, which then exits the catalyst surface. A similar process repeats until the other two H_2_ molecules are produced (Figure 8) [96–97].

**Figure 9:**
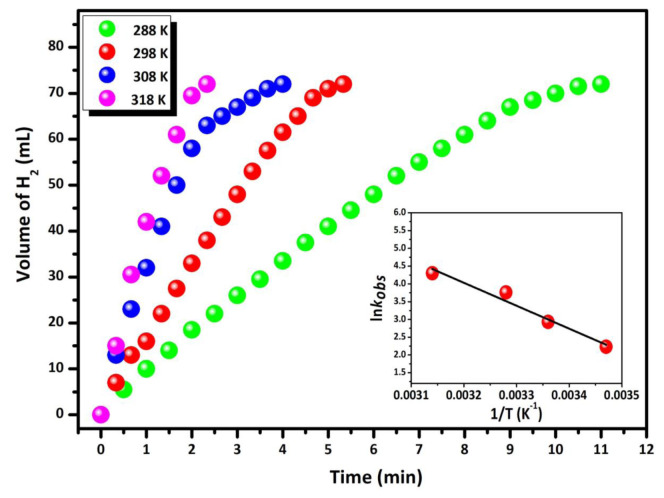
Graphs of the gas volume vs. time for the methanolysis of HB starting with 200 mM HB plus 100.0 mg Pd/La_2_O_3_ catalyst (4.69 mM) at different temperatures (288, 298, 308, and 318 K).

In catalytic methanolysis reactions with an HB concentration of 200 mM and a Pd/La_2_O_3_ catalyst amount of 100 mg (4.69 mM), the effect of temperature changes (288, 298, 308, and 318 K) on the H_2_ formation rate was measured at regular intervals. The volume/time graph obtained is shown in Figure 9. Upon examination of the obtained graph, it is observed that catalytic methanolysis is completed rapidly, even at temperatures below room temperature, and the H_2_ formation rate changes in direct proportion to the temperature. For the methanolysis of HB catalyzed by the Pd/La_2_O_3_ catalyst, activation parameters such as *E*_a_, *ΔH^#^*, and *ΔS^#^* were calculated from temperature-dependent graphs drawn using Arrhenius and Eyring kinetic equations (Figures S2 and S3). The slope of the linear part of each hydrogen generation versus time plot at different temperatures indicates the reaction rate constant kobs of the methanolysis reaction. While the Arrhenius curve (Figure 9, inset) was used for the activation energy (*E*_a_), which was found to be 53.66 kJ/mol (Figure S2), the Eyring curve was used to calculate the activation enthalpy (*ΔH^#^* = 51 kJ/mol) and activation entropy (*ΔS^#^* = 48.5 J/mol×K) values (Figure S3). This *E*_a_ value is the lowest calculated Ea for HB methanolysis compared to the catalytic systems summarized in Table.

**Figure 10:**
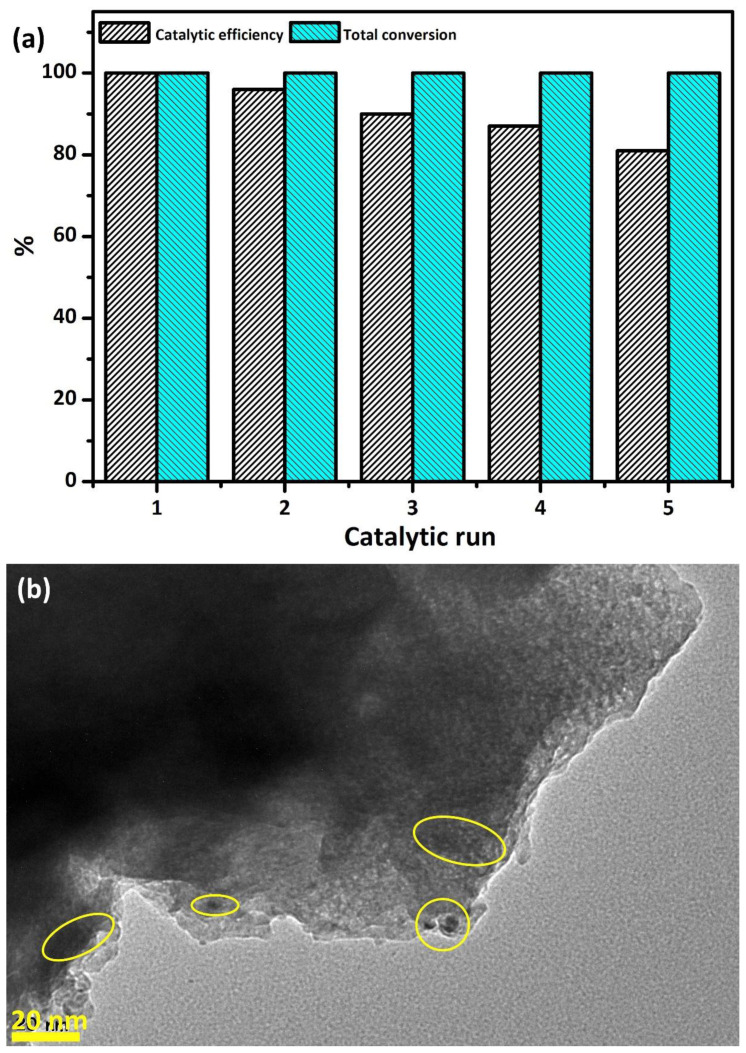
(a) Time plots of catalytic methanolysis of HB by up to 5 recycle for the Pd/La_2_O_3_ catalyst at 298 K and, (b) TEM image of Pd/La_2_O_3_ catalyst obtained after the 5th catalytic recycle.

After detailed kinetic studies, the recyclability performance of the Pd/La_2_O_3_ catalyst in hydrogen production from HB methanolysis was investigated (Figure 10). The study aimed to determine the catalytic recycling durability of the prepared Pd/La_2_O_3_ catalyst, when all of the HB was converted to the product, more HB was added to the solution medium and the catalyst efficiency in repeated catalytic reactions was determined. At the end of the methanolysis reactions continued for up to 5 cycles, it was determined that the Pd/La_2_O_3_ catalyst exhibited extremely high stability and durability, preserving 81% of the initial yield and achieving approximately 99% conversion even in the 5th cycle. Some reduction in catalytic activity can be attributed to the closure of the catalyst’s active surfaces by methanolysis reaction products or to the increase in particle size or aggregation of Pd (0) NPs. This is also evident in the TEM image of the product isolated as a result of the 5th catalytic cycle, with clusters circled in Figure 10b.

## 4. Conclusions

The prepared Pd/La_2_O_3_ catalyst yielded significant results in the hydrogen production from HB methanolysis:

- Using an easy and reproducible impregnation-reduction technique, we synthesized Pd(0) NPs supported on La_2_O_3_;
- To elucidate the structural and morphological characteristics of Pd/La_2_O_3_ catalyst, we employed SEM, SEM-Elemental Mapping, XRD, XPS, ICP-OES, TEM, HRTEM and TEM-EDX techniques;
- Compared with the initial turnover frequency of the second heterogeneous system used for the HB methanolysis, the Pd/La_2_O_3_ catalyst exhibits a catalytic efficiency of 24.4 min^−1^ at 298 K;
- The properties of Pd/La_2_O_3_, characterized by high recyclability and excellent resistance to aggregation, have been verified. The catalyst demonstrates remarkable stability during catalytic recycles, retaining 81% of the initial yield and exceeding 99% in the 5th recycling;
- It has been demonstrated that the methanolysis of HB in the presence of the Pd/La_2_O_3_ catalyst follows second and ≈first-order kinetics with respect to Pd and HB concentrations, respectively. This conclusion is drawn from detailed kinetic studies that are dependent on Pd/La_2_O_3_ catalyst and HB concentrations. For the HB methanolysis catalyzed by the Pd/La_2_O_3_ catalyst, temperature-dependent kinetic studies were conducted, leading to anticipated values for the activation energy (*E_a_*), enthalpy of activation (*ΔH^#^*), and entropy of activation (*ΔS^#^*) parameters (*E_a_* = 53.66 kJ/mol, *ΔH^#^* = 51 kJ/mol, *ΔS^#^* = 48.5 J/mol×K). Notably, the calculated *E_a_* value is the lowest reported for HB methanolysis compared to other catalytic systems in the literature.

## Supplementary Material

### Materials

Palladium (II) nitrate dihydrate (Pd(NO_3_)_2_·2H_2_O), lanthanum (III) oxide (La_2_O_3_), hydrazine hemisulfate salt (H_2_NNH_2_·0.5H_2_SO_4_, ≥98 %), methanol (CH_3_OH, 99 %), tetrahydrofuran (C_4_H_8_O, THF, 99.9 %) and sodium borohydride (NaBH_4_, powder, ≥98.0%) purchased from Sigma-Aldrich were used as-received and without further purification. Under argon atmosphere methanol and THF were distilled using sodium-benzophenone and magnesium. A water purification system for distilled deionized water was used (Milli-Q Water Purification System). The catalytic materials isolated at the end of the synthesis were stored in a in vacuum atmosphere. Acetone was used to wash all glassware and Teflon-coated magnetic stir sticks and rinsed profusely with distilled water before drying in an oven at 393 K.

#### Synthesis of hydrazine-borane (N_2_H_4_BH_3_, HB)

The synthesis of HB was performed as described in the literatureS1–S2. According to the reaction in Scheme S1, the preparation of the HB is carried out according to the reaction of sodium borohydride with hydrazine hemisulfate salt in THF at 25 °C. The purity of the hydrazine-borane used in the experiments was determined using FT-IR, ^1^H, and melting point analytical methods. Melting point of HB: 60.6 °C; ^1^H-NMR (400 MHz, CH_2_Cl_2_-d_2_) 5.1 ppm (t, 2, N_2_H_4_BH_3_), 3.4 ppm (b, 2, N_2_H_4_BH_3_), 1.2 ppm (t, 3, N_2_H_4_BH_3_); FT-IR (selected, cm^−1^) 3333 (s), 3220 (s), 2836 (m), 2662 (m), 2367 (m), 1622 (s), 1592 (m), 1437 (w), 1338 (m), 1157 (s), 912 (m), 750 (w). The obtained results are consistent with those of previous studiesS1–S3.

#### Characterization techniques

The crystal information of the obtained materials was gained with a Rigaku Ultima-IV model X-ray diffraction (Cu K_a_ radiation, λ = 1.54 Å). The working voltage and current of the X-ray tube were 40 kV and 40 mA, respectively. The surface morphology and microstructure of the obtained catalysts were observed by using TEM and HRTEM techniques. TEM and HRTEM samples were prepared by dropping one drop of dilute suspension on a copper-coated carbon TEM grid and then drying the solvent. TEM analysis was carried out on a JEOL JEM-200CX transmission electron microscope operating at 120 kV. HRTEM analyses were run on a JEOL JEM-2010F transmission electron microscope operating at 200 kV. The 3D morphological images of samples were carried out using a Zeiss Sigma 300 model field emission scanning electron microscope (FESEM) with an energy dispersive X-ray (EDX) spectroscopy and In-Lens (SE1) detector at 10kV accelerating voltage. The surface chemistry information of the obtained catalysts was acquired on a Kratos AXIS Ultra spectrometer was employed for XPS analysis using monochromatic Al-Kα radiation (1486.6 eV, the X-ray tube working at 15 kV and 350 W, and pass energy of 23.5 eV). The C 1s photoelectron line (binding energy = 284.6 eV) was used to calibrate the binding energies of the photoelectron. The metal content in the obtained catalysts was evaluated with the inductively coupled plasma optical emission spectrometry (ICP-OES) analysis on a Perkin Elmer Optima 4300DV ICP emission spectrometer. Before ICP-OES analysis, each sample was completely dissolved in a mixture of HNO_3_/HCl (1/3; v/v). Fourier-transform infrared (FT-IR) spectra were taken on a Bio-Rad-Win-IR spectrophotometer carrying out KBr discs in between 4000 and 400 cm^−1^. NMR instrument (Bruker 400 MHz Ultrashield TM) for NMR analysis was used, which is applied deuterated dichloromethane (CH_2_Cl_2_-d_2_) as solvent using tetramethylsilane (TMS) as internal standards.

Figure S1 Graphs of released volume of gas vs. time for 200 mM HB methanolysis with La_2_O_3_ and Pd/La_2_O_3_ catalyst at 298 K.

Figure S2 Arrhenius curve.

Figure S3 Eyring curve.

Scheme S1 Synthesis protocol of HB in THF at 25 °C.

## Figures and Tables

**Scheme:**
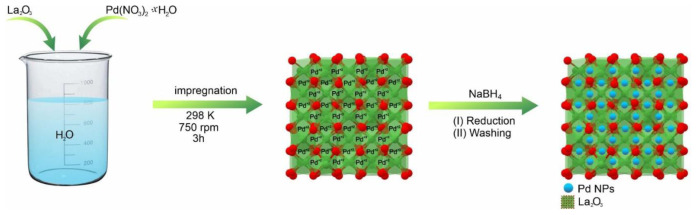
Schematic presentation of the synthesis of Pd/La_2_O_3_ catalyst.

**Table:** Comparing thermodynamic parameters and activities of catalysts used in the formation of H_2_ as a result of HB methanolysis.

Catalyst	TOF*a*	E_a_*b*	T (K)	Ref.
**NiCl** **_2_**	24.0	65	298	[93]
**PVP-stabilized Ni NPs**	35.6	63 ± 2	298	[50]
**PdCl** **_2_**	NG	100.3	303	[94]
**Ru NPs@** ***nano*** **-CeO** **_2_**	41.05	102.6	298	[44]
**Pd/La** **_2_** **O** **_3_**	**24.4**	**53.7**	**298**	**This study**

a Turnover frequency, mol H_2_ mol cat^−1^ min^−1^;

b Activation energy (kJ/mol), NG (not given), PVP, polyvinylpyrrolidone.
