# Supplementary figures and images for: Palladium (0) nanoparticles distributed on lanthanum (III) oxide as an effective catalyst for the methanolysis of hydrazine-borane to produce hydrogen
Source: Turk J Chem. 2024 Jan 3;48(1):137–51. doi: 10.55730/1300-0527.3646 (PMC10965170; doi:10.55730/1300-0527.3646)

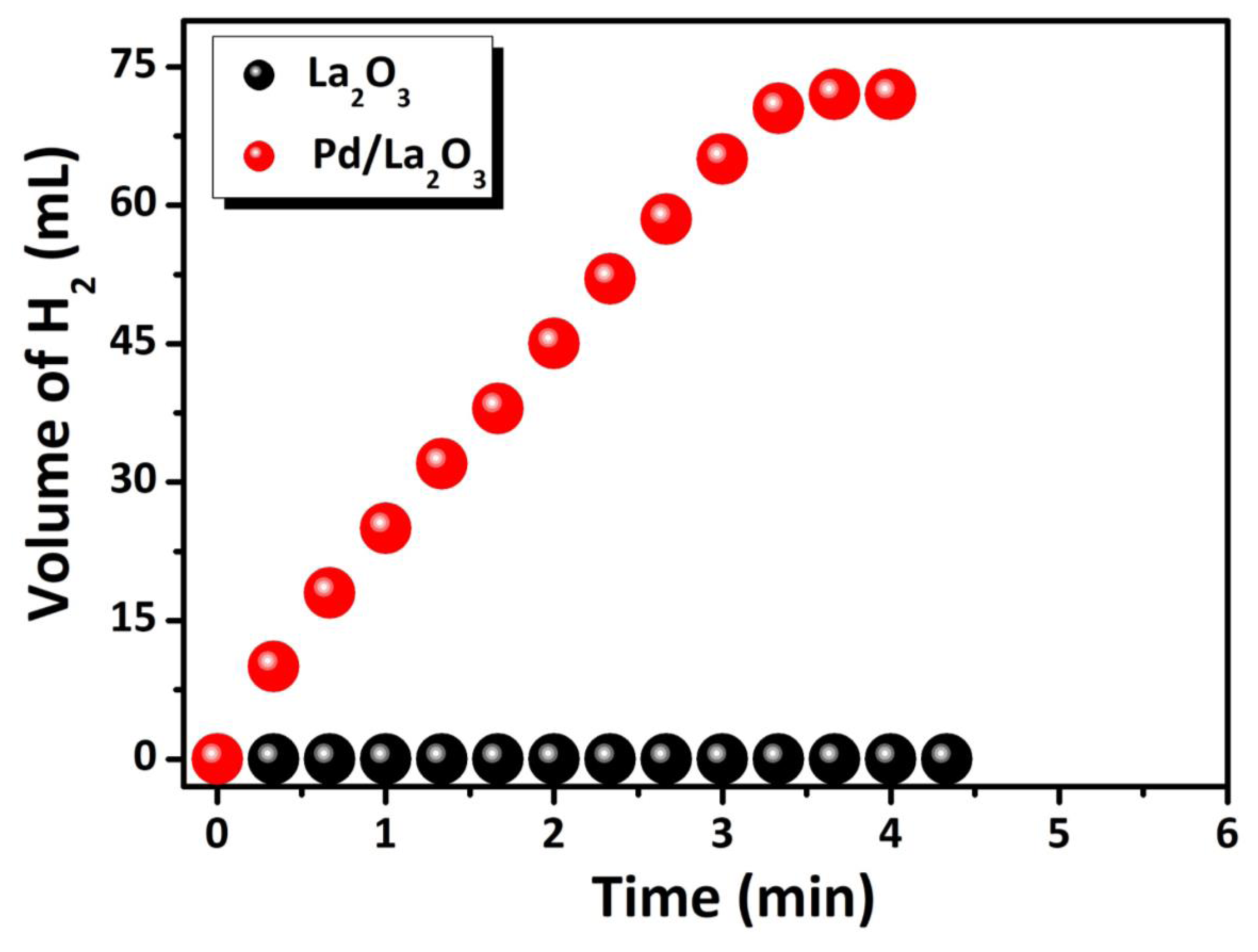

Supplement: Figure S1 — Graphs of released volume of gas vs. time for 200 mM HB methanolysis with La2O3 and Pd/La2O3 catalyst at 298 K. [file tjc-48-01-0137s1.tif]

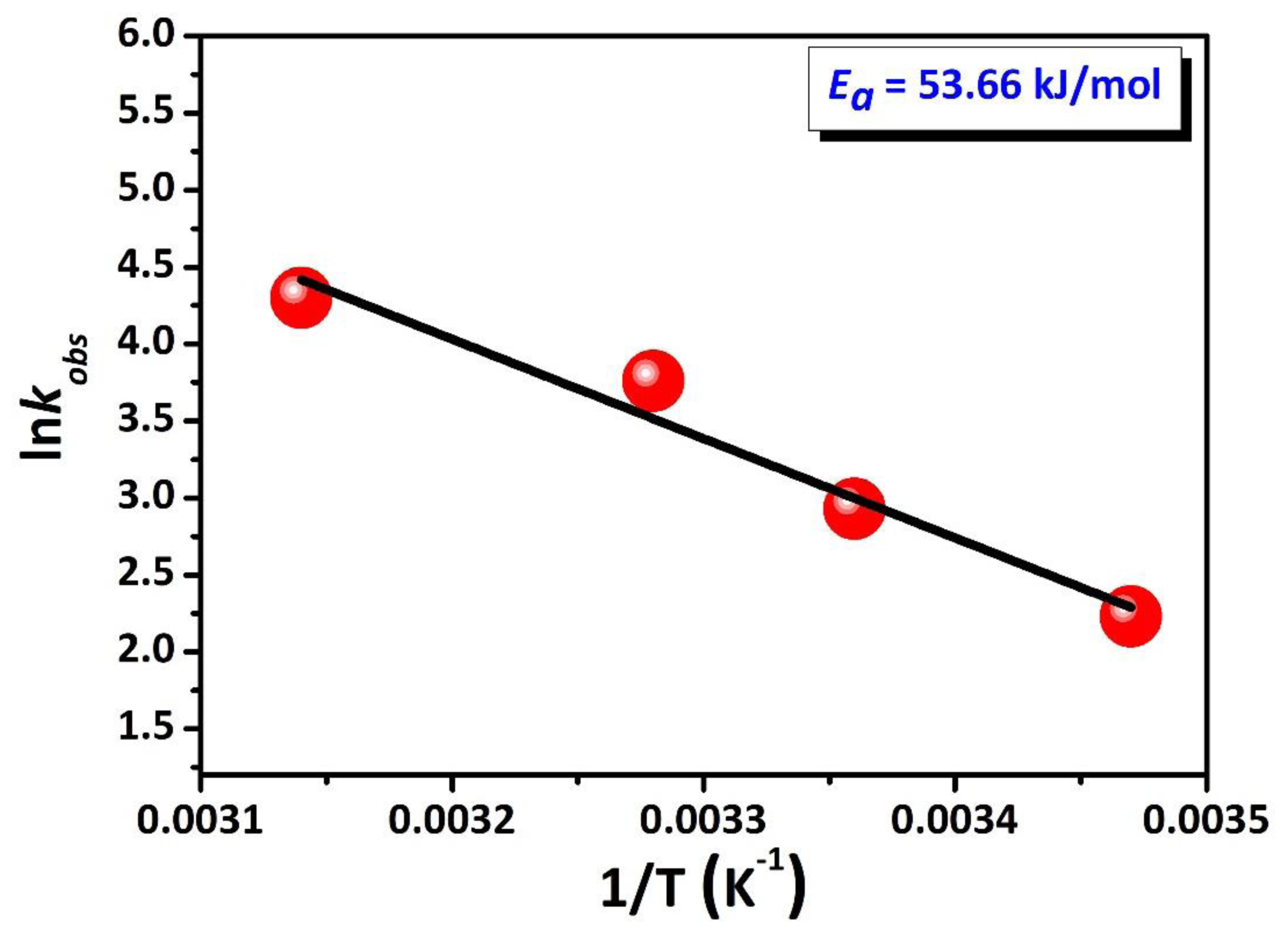

Supplement: Figure S2 — Arrhenius curve. [file tjc-48-01-0137s2.tif]

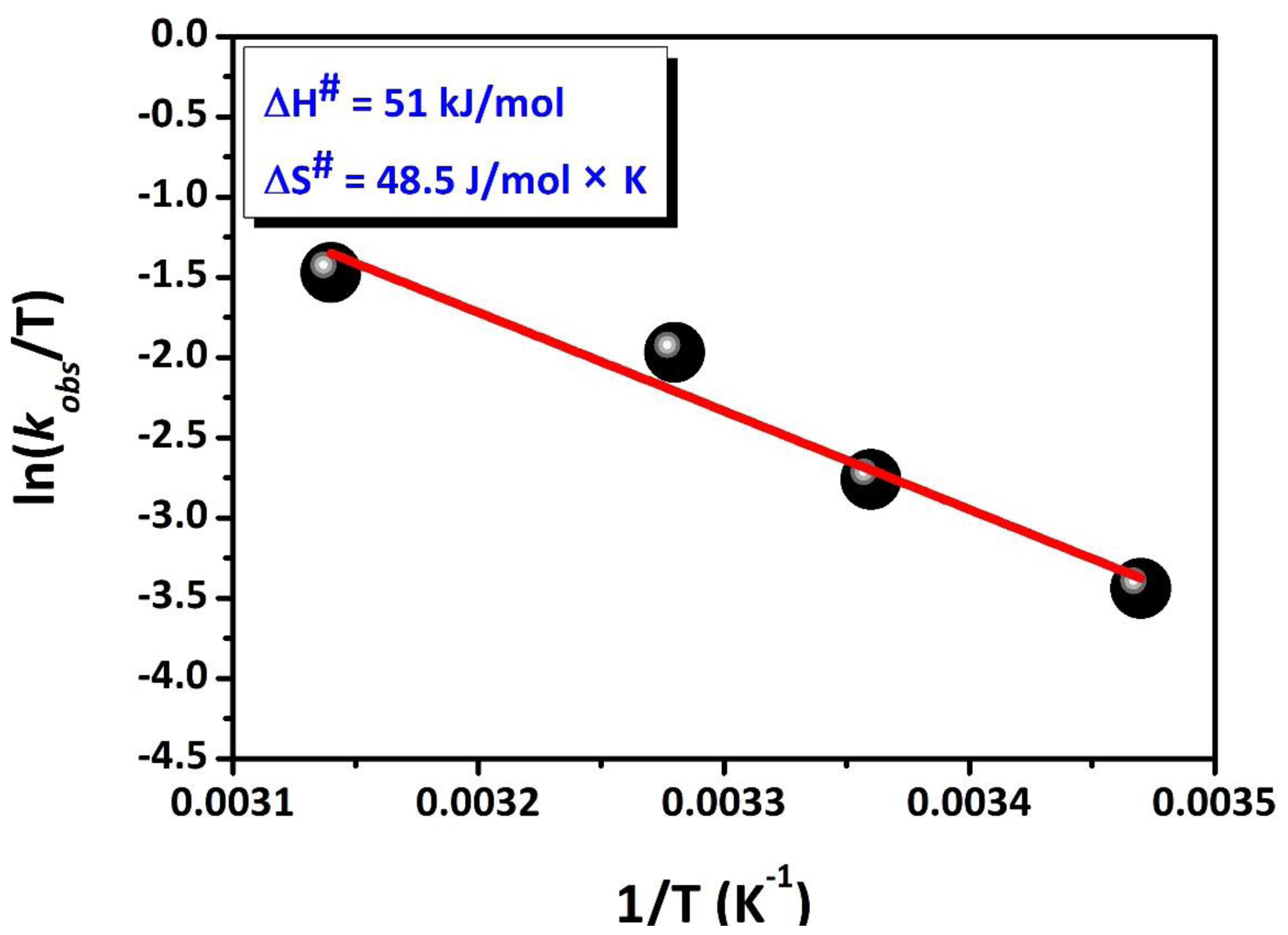

Supplement: Figure S3 — Eyring curve. [file tjc-48-01-0137s3.tif]

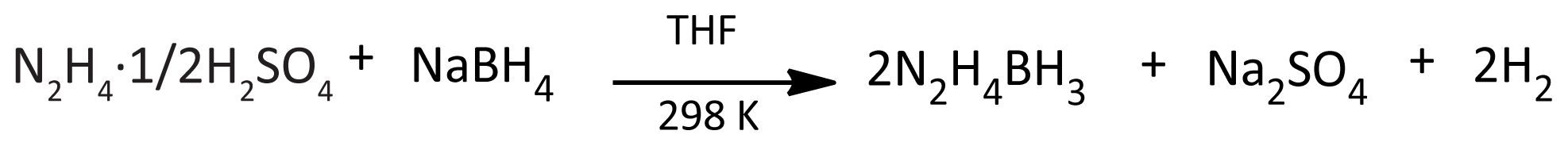

Supplement: Scheme S1 — Synthesis protocol of HB in THF at 25 °C. [file tjc-48-01-0137s4.tif]
